# Analysis of Neurodevelopment in Children Born Extremely Preterm Treated With Acid Suppressants Before Age 2 Years

**DOI:** 10.1001/jamanetworkopen.2022.41943

**Published:** 2022-11-15

**Authors:** Elizabeth T. Jensen, Joe Yi, Wesley Jackson, Rachana Singh, Robert M. Joseph, Karl C. K. Kuban, Michael E. Msall, Lisa Washburn, Rebecca Fry, Andrew M. South, T. Michael O’Shea

**Affiliations:** 1 Department of Epidemiology and Prevention, Wake Forest University School of Medicine, Winston-Salem, North Carolina; 2 Department of Biostatistics, Gillings School of Global Public Health, University of North Carolina at Chapel Hill, Chapel Hill; 3 Department of Pediatrics, University of North Carolina School of Medicine, Chapel Hill; 4 Department of Pediatrics, Tufts University School of Medicine, Boston, Massachusetts; 5 Department of Anatomy and Neurobiology, Boston University School of Medicine, Boston, Massachusetts; 6 Department of Pediatrics, Boston Medical Center, Boston; 7 Kennedy Research Center on Intellectual and Neurodevelopmental Disabilities, University of Chicago Pritzker School of Medicine, Chicago, Illinois; 8 Department of Pediatrics, Wake Forest University School of Medicine, Winston-Salem, North Carolina

## Abstract

**Importance:**

Children born preterm are at increased risk of adverse neurodevelopmental outcomes and may be particularly vulnerable to the effects of gastric acid suppression during infancy.

**Objective:**

To assess whether early acid suppressant use in infants born extremely preterm is associated with poorer neurodevelopmental outcomes.

**Design, Setting, and Participants:**

The Extremely Low Gestational Age Newborn study was a multicenter, longitudinal cohort study of infants born before 28 weeks’ gestational age between March 22, 2002, and August 31, 2004. The current analyses were performed from September 12, 2020, through September 22, 2022. Of the 1506 infants enrolled, 284 died before discharge and 22 died before 24 months of age. An additional 2 died before age 10 years, leaving 1198 (79.5%) eligible for a visit. Of these, 889 (74%) participated in the visit at age 10. At age 10 years, the association of early-life acid suppressant use with neurocognitive, neurodevelopmental, and psychiatric symptomatology was assessed.

**Exposures:**

Acid suppressant use before 24 months of age was determined from medical records and from questionnaires administered to mothers.

**Main Outcomes and Measures:**

Neurodevelopmental assessments at age 10 years included the School-Age Differential Ability Scales–II, the Developmental Neuropsychological Assessment-II, the Autism Diagnostic Observation Schedule-2, the Social Responsiveness Scale-2, and the Child Symptom Inventory-4 for attention-deficit/hyperactivity disorder (ADHD), depression, and anxiety.

**Results:**

Of the 889 participants assessed at age 10 years (mean [SD] age, 9.97 [0.67] years; mean [SD] gestational age at birth, 26.1 [1.3] weeks; 455 [51.2%] male), 368 (41.4%) had received acid suppressants by 24 months of age. Associations were observed between acid suppressant use and decreased full-scale IQ *z* score (adjusted β, −0.29; 95% CI, −0.45 to −0.12), verbal IQ *z* score (adjusted β, −0.34; 95% CI, −0.52 to −0.15), nonverbal IQ *z* score (adjusted β, −0.22; 95% CI to −0.39 to −0.05), working memory *z* score (adjusted β, −0.26; 95% CI to −0.45, −0.08), autism spectrum disorder (adjusted relative risk, 1.84; 95% CI, 1.15-2.95), and epilepsy (adjusted relative risk, 2.07; 95% CI, 1.31 to 3.35). Results were robust to multiple sensitivity analyses. Use of acid suppressants was not associated with inhibitory control, ADHD, anxiety, or depression.

**Conclusions and Relevance:**

The results of this cohort study suggest that early-life use of acid suppressants in extremely preterm infants may be associated with poorer neurodevelopmental outcomes and add to evidence indicating caution in use of these agents.

## Key Points

**Question:**

Is use of acid suppressants in infants born extremely preterm associated with an increased risk of poor neurodevelopmental outcomes?

**Findings:**

In this cohort study of 889 children born extremely preterm who were assessed at age 10 years, acid suppressant use in the first 24 months of life was associated with decreased IQ and impaired working memory, as well as an increased risk of autism spectrum disorder. Use of acid suppressants was not associated with inhibitory control, attention-deficit/hyperactivity disorder, anxiety, or depression.

**Meaning:**

The results of this study suggest that acid suppressant use in extremely preterm infants may have long-term neurodevelopmental consequences and add to evidence indicating need for caution in use of these agents.

## Teaser

This cohort study assessed neurodevelopmental outcomes at age 10 years in children who were born extremely preterm and received acid suppressants in the first 24 months of life.

## Introduction

Despite efforts to limit use of acid suppressants in infants,^1,2,3,4^ use of these medications, including both proton pump inhibitors (PPIs) and histamine 2 receptor antagonists (H2RAs), remains prevalent among both term and preterm infants.^5,6,7^ Relative to term-born infants, those born preterm are treated more often with acid suppressants, with a prevalence of nearly 30% for H2RAs and nearly 20% for PPIs during neonatal intensive care unit (NICU) hospitalization.^8^ Further, many preterm-born infants are first exposed to these medications after NICU discharge.^9,10^ Although acid suppressants are prescribed for a variety of indications^2^ and prophylactically,^7^ numerous publications have questioned the benefit of acid suppressant use in children, particularly for the treatment of gastroesophageal reflux.^11,12,13,14^ Of particular concern are studies suggesting an association between acid suppressant use and risk of atopy, obesity, bone fractures, and infections in children.^14,15,16,17,18,19,20,21^ Among children born preterm, long-term outcomes have not been reported, but an association with an increased risk of death has been reported.^22^

In murine models, administering acid suppressants in the setting of acute illness has resulted in differential expression of genes involved in behavioral and cognitive pathways, with differential expression of genes corresponding to the relative abundance of certain microbial families.^23^ Acid suppressants have been found to be associated with altered microbiome colonization and dysbiosis.^23,24,25,26^ Preterm infants, given their neurodevelopmental immaturity at birth,^27^ may be especially vulnerable to deleterious effects of acid suppressants. Here we analyzed data from the Extremely Low Gestational Age Newborns (ELGAN) Study to evaluate the association between PPI and H2RA use during infancy and neurodevelopmental outcomes in later childhood, including intellectual ability (IQ), executive function (EF), epilepsy, social ability, autism spectrum disorder (ASD), attention-deficit/hyperactivity disorder (ADHD), and symptoms of anxiety and depression.

## Methods

### Participants

Begun in 2002, the ELGAN Study is an ongoing multicenter, prospective observational birth cohort investigating the risks of structural and functional neurological disorders in individuals born extremely preterm (<28 weeks’ gestational age).^28^ The present study reflects a secondary analysis of the data collected in the ELGAN study. Infants were enrolled between March 22, 2002, and August 31, 2004. Of the 1506 infants enrolled, 284 died before discharge and 22 died before 24 months of age; an additional 2 died before age 10 years, leaving 1198 (79.5%) eligible for a visit, when 889 (74.7% of the 1198) participated in neurocognitive and behavioral assessments. The current analyses were performed from September 12, 2020, through September 22, 2022. The institutional review boards of all participating institutions approved the study procedures, and written informed consent and assent were obtained. This study followed the Strengthening the Reporting of Observational Studies in Epidemiology (STROBE) reporting guideline for cohort studies.

### Demographic and Newborn Characteristics

Demographic data (including sex, maternal race and ethnicity, maternal educational level, and receipt of public insurance [yes or no]) were collected from the mother by a research nurse using a structured data collection form and defined interview process. Race and ethnicity were not included in our adjusted models; we used a directed acyclic graph (eFigure in the Supplement) to define the set of covariates and intentionally tried to include other factors often associated with race but better markers of disparity (eg, receipt of insurance and educational level). Estimates of the gestational age at delivery were based on a hierarchy of the quality of available information.^28^ Data on infant clinical attributes during NICU admission were collected through abstraction of data from the medical record and through brain ultrasonographic assessment for white matter damage, performed as a component of standard care. For the current analysis we used data from ultrasonographic scans obtained in the entire range between birth and 40 weeks’ postmenstrual age. For the current study, we defined white matter damage as the presence of either parenchymal echolucency (hypoechoic lesion), echodensity (hyperechoic lesion), or moderate to severe ventriculomegaly on a late scan (performed after the first 2 postnatal weeks).^29^ Postdischarge clinical attributes, including infant feeding difficulties, cerebral palsy diagnosis, and use of a feeding tube were obtained from questionnaires administered to the mothers and clinical assessments completed at 12 and 24 months.^30,31^

### Acid Suppressant Use

Data on acid suppressant use during NICU hospitalization were obtained through abstraction of medical records and at 12 and 24 months’ corrected age from parent surveys. Any documentation of use of a PPI or H2RA within the first 24 months of life constituted early-life exposure to acid suppressants. Documentation of medications was reviewed individually (E.T.J., J.Y., and T.M.O.) to ensure that all generic and branded medications were identified.

### Neurocognitive Function

Neurocognitive assessments were conducted with validated instruments administered by child psychologists (including R.M.J.) who underwent in-person training and certification for administration and scoring and were not aware of participants’ medical histories or medication use. General cognitive ability (or IQ) was assessed with the School-Age Differential Ability Scales–II (DAS-II) Verbal and Nonverbal Reasoning scales. Executive function was assessed with the DAS-II and the Developmental Neuropsychological Assessment, second edition (NEPSY-II). Verbal working memory was measured using the DAS-II Recall of Digits–Backward and Recall of Sequential Order subtests. Simple inhibition and inhibition in the context of set shifting were assessed using the NEPSY-II Inhibition Inhibition and Inhibition Switching subtests, respectively.

Latent profile analysis (LPA) was used to classify participants into 4 subgroups based on similarities in their profiles of verbal and nonverbal IQ and EF scores from the DAS-II and NEPSY-II^32^: (1) normal, characterized by mean IQ and EF scores within normal range on all measures; (2) low-normal, characterized by mean IQ and EF scores ranging from 0.5 to more than 1 SD below the norm; (3) moderately impaired, characterized by mean IQ and EF measures between 1.5 and 2.5 SD below the norm; and (4) severely impaired, characterized by mean IQ and EF measures 3 to 4 SD below the norm.^32^ Low-normal and normal were combined and moderately impaired and severely impaired were combined for a binary composite of neurocognitive function.

### Neurodevelopmental Disorders

Neurodevelopmental disorders assessed included epilepsy, ASD, non-ASD social disability, and ADHD. Identification of epilepsy included a parent-completed validated seizure screening, followed by a structured interview with a study coordinator and an open-ended interview with a pediatric epilepsy specialist.^33^ A second, and when necessary a third, epilepsy specialist (K.C.K. was among these) reviewed interview responses for consistency. We defined epilepsy as having 2 or more unprovoked seizures.

Diagnostic assessment of ASD was conducted with 3 validated measures administered sequentially. Children determined to be at risk on the Social Communication Questionnaire were assessed with the Autism Diagnostic Interview–Revised (ADI-R), an in-depth parent interview. Children meeting ADI-R criteria for ASD were administered the Autism Diagnostic Observation Schedule-2 (ADOS-2). Children meeting standardized research criteria for ASD on the ADOS-2 were classified as having ASD.^34,35^

Social ability was assessed using the Social Responsiveness Scale-2 (SRS-2) Social Communication and Interaction (SCI) and Restricted Interests and Repetitive Behavior (RRB) T scores (T scores are standardized scores with a normative mean of 50 and an SD of 10).^36^ An ADHD diagnosis was based on the Child Symptom Inventory-4 (CSI-4),^37^ completed by parents and the child’s teacher, and parental report of an ADHD diagnosis by a physician. Children were classified as having ADHD if this diagnosis was supported by at least 2 of the 3 sources of information.^38,39^

### Psychiatric Symptoms of Anxiety and Depression

Anxiety and depression symptoms were assessed via parent report on the CSI-4.^37^ An average of T scores for parent-reported generalized anxiety disorder, social phobia, and separation anxiety was used to represent global anxiety symptoms.^40^ Similarly, T scores on major depressive disorder and dysthymic disorder subscales were averaged to represent depression symptoms.

### Statistical Analysis

#### Primary Analyses

Missing data were examined and at age 10 years; 10.5% of participants were missing data on feeding difficulties in early life and 0.8% were missing data for chronic lung disease. Multiple imputation models were constructed to impute missing covariate data and preserve the full sample at age 10. We examined the distribution of acid suppressant use according to study outcomes and modeled acid suppressant use according to documentation of any use of an acid suppressant in early life (during NICU hospitalization or after NICU discharge and within the first 24 months of life) vs no documentation of use in early life (reference). Continuous outcomes were estimated using linear regression models, and dichotomous outcomes were estimated using Poisson regression models with robust SEs for estimating the crude and adjusted relative risk (aRR) with 95% CIs of a given outcome conditional on acid suppressant use in early life. Generalized estimating equations with exchangeable correlation matrices were used to account for lack of independence from multiparous births. Directed acyclic graphs^41^ were used to inform selection of possible confounders for inclusion in adjusted models (eFigure in the Supplement). These included neonatal cerebral white matter damage (yes vs no [reference]); feeding difficulties as reported by the mother on the 12-month survey (yes vs no [reference]); maternal insurance status as reported by the mother during birth hospitalization (private or other vs public insurance [reference]); chronic lung disease, defined as having received supplemental oxygen at 36 weeks’ postmenstrual age (yes vs no [reference]); maternal education as reported on the maternal interview at enrollment (some college or college graduate and higher vs ≤ high school diploma [reference]), and infant sex.

#### Secondary Analyses

In a secondary analysis of associations observed between acid suppressant use and ASD, we further delineated ASD into children with and without intellectual impairment, as defined by a nonverbal IQ of less than 70 on the DAS-II.^42,43^ We used Poisson models to estimate the association of acid suppressant use and ASD subtype (ASD with intellectual impairment, ASD without intellectual impairment, no ASD but intellectual impairment, and no ASD and no intellectual impairment [reference]).

#### Sensitivity Analyses

In sensitivity analyses, to evaluate the robustness of our results, we performed multinomial regression, modeling the association between early-life acid suppressant use and 2 levels of impairment (ie, *z* score less than or equal to −2 and *z* score greater than −2 to greater than or equal to −1, with *z* score greater than −1 and less than 1 as reference group) for DAS-II verbal and nonverbal IQ, DAS-II Working Memory, and NEPSY-II Inhibition Inhibition and Inhibition Switching. Next, to assess whether model fit was improved by independently assessing PPIs and H2RAs, we performed a likelihood ratio test of the nested models with and without additional parameterization of acid suppressant type (H2RA vs PPI). Between NICU discharge and follow-up at age 10 years, the proportion of mothers with lower education and from underrepresented racial and ethnic groups decreased (eTable 1 in the Supplement). To evaluate the potential for selection bias due to loss to follow-up, we examined the distribution of demographic and clinical factors believed a priori to be associated with neurodevelopmental outcomes across follow-up, comparing distributions of variables collected at NICU discharge and at age 10 years. Given observations of differential loss to follow-up for factors potentially associated with study outcomes and the potential for confounding by indication, we used an inverse probability weighting (IPW) model, jointly modeling the probabilities of exposure to an acid suppressant and of staying in the study and applying the inverse probability weights to the existing models to estimate associations between acid suppression use and study outcomes (cognitive function LPA classification, ASD, epilepsy, ADHD, anxiety, and depression). Further, we performed a propensity score–matched analysis to address the potential for confounding by indication. Here, an optimal matching algorithm was computed from potential confounders (maternal education, public insurance, white matter damage, chronic lung disease, feeding problems at age 1 year, and infant sex) of both exposure and neurocognitive outcomes. Propensity scores were calculated and the optimal match for each control was selected based on the overall difference in the likelihood of exposure. We also modeled exposure according to timing of exposure (NICU only, both NICU and postdischarge use, and postdischarge use only). Finally, we conducted analyses restricted to a participants without any diagnosis of cerebral palsy and also with use of a feeding tube reported.

All analyses were performed using SAS software, version 9.4 (SAS Institute Inc). Statistical significance was defined as a 95% CI excluding 0 for beta coefficients and 1 for relative risks.

## Results

### Primary Analyses

Of the initial 1506 enrolled, 1198 survived to age 10 years (mean [SD] gestational age at delivery, 26.1 [1.3] weeks; 621 [51.8%] male and 577 [48.2%] female). Among 1179 mothers, 322 (27.3% ) were Black, 706 (59.9%) were White, and 151 (12.8%) were categorized as other (including Asian, American Indian, mixed race, and other race; these groups were not analyzed individually because data were too sparse for further analysis; 147 [12.3%] of 1192 mothers were Hispanic, and 1045 [87.7%] were non-Hispanic) (denominators for maternal race and ethnicity reflect missing responses) (Table 1, eTable 1 in the Supplement).

**Table 1.** Demographic and Clinical Characteristics of 1198 Participants Who Survived to Age 24 Months According to Acid Suppressant Use in Early Life^a^

Characteristic	Participants, No. (%)	
	No acid suppressant use (n = 712)	Acid suppressant ≤24 mo of age (n = 486)
Gestational age at delivery, mean (SD), wk	26.2 (1.2)	26.0 (1.3)
Sex		
Male	343 (48.2)	278 (57.2)
Female	369 (51.8)	208 (42.8)
Weight for gestational age at delivery, *z* score, mean (SD)	−0.15 (1.0)	−0.19 (1.1)
Chronic lung disease		
No oxygen or mechanical ventilation at 36 wk	383 (53.8)	207 (42.6)
Oxygen at 36 wk	275 (38.6)	223 (45.9)
Mechanical ventilation at 36 wk	45 (6.3)	55 (11.3)
White matter damage	133 (18.7)	109 (22.4)
Maternal race (n = 1179)^b^		
Black	220 (30.9)	102 (21.0)
White	402 (56.5)	304 (62.6)
Other^c^	79 (11.1)	72 (14.8)
Maternal ethnicity (n = 1192)^b^		
Hispanic	77 (10.8)	70 (14.4)
Non-Hispanic	631 (88.6)	414 (85.2)
Maternal education, y (n = 1152)^b^		
≤12	302 (42.4)	204 (42.0)
>12 to <16	168 (23.6)	102 (21.0)
≥16	224 (31.5)	152 (31.3)
Public insurance (n = 1173)^b^		
No	421 (59.1)	288 (59.3)
Yes	282 (39.6)	182 (37.4)
Feeding difficulties at 12 mo (n = 1019)^b^		
No	461 (64.8)	285 (58.6)
Yes	144 (20.2)	129 (26.5)

^a^ Sample that survived to age 24 months and included in inverse probability weighted analyses.

^b^ Responses did not sum to 1198 because of missing responses.

^c^ Other included 28 (2.4%) Asian, 12 (1.0%) American Indian, 35 (3.0%) of mixed race, and 76 (6.4%) participants who indicated other on the questionnaire and for whom the racial group could not be determined; these groups were not analyzed individually because data were too sparse for further analysis (data were missing for 19 mothers because no response was given); 147 (12.3%) of 1192 mothers were Hispanic, and 1045 (87.7%) were non-Hispanic (data were missing for 6 mothers because no response was given).

Of these, 889 participants (74.2%) were assessed at age 10 years (mean [SD] age, 9.97 [0.67] years; mean [SD] gestational age at birth, 26.1 [1.3] weeks; 455 [51.2%] male, 434 [48.8%] female) (eTable 1 in the Supplement). Of the 889 participants, 368 (41.4%) received an acid suppressant within the first 24 months of life (256 [28.8%] in the NICU and 112 [12.6%] after NICU discharge). The demographic distribution of those exposed and unexposed to acid suppressants was generally similar, but those exposed to acid suppressants were more likely to have required mechanical ventilation at 36 weeks (55 [11.3%] vs 45 [6.3%]), were more likely to have white matter damage documented (109 [22.4%] vs 133 [18.7%]), and were more likely to have experienced feeding difficulties at 12 months of age (129 [26.5%] vs 144 [20.2%]) (Table 1).

Acid suppressant use was associated with reduced full-scale IQ (adjusted β, −0.29; 95% CI, −0.45 to −0.12; this equated to a decrease of 4.35 [95% CI, 6.75-1.80] points on full scale IQ), reduced verbal IQ (adjusted β, −0.34; 95% CI, −0.52 to −0.15), and reduced nonverbal IQ (adjusted β, −0.22; 95% CI, −0.39 to −0.05) (Table 2). Children who used an acid suppressant also had reduced scores for DAS-II Working Memory (adjusted β, −0.26; 95% CI, −0.45 to −0.08) and Inhibition Inhibition (adjusted β, −0.22; 95% CI, −0.38 to −0.05). We observed no association between acid suppressant use and Inhibition Switching subtests. For the LPA-derived classifications of cognitive function, the risk of moderate to severe impairment, relative to low-normal or normal cognitive function, was higher for children who used an acid suppressant in early life (aRR, 1.40; 95% CI, 1.13-1.74).

**Table 2.** Acid Suppressant Use and Neurocognitive, Neurodevelopmental, and Psychiatric Outcomes Among 889 Children Assessed at Age 10 Years

Outcomes	Acid suppressant use at ≤24 mo of age, No. (%)		*z* Score		RR (95% CI)	aRR (95% CI)^a^
	None	Any	β (95% CI)	Adjusted β (95% CI)^a^		
**DAS-II verbal IQ**						
≤−2	70 (13.6)	80 (22.4)	−0.43 (−0.63 to −0.23)	−0.34 (−0.52 to −0.15)	NA	NA
>−2 to ≤−1	94 (18.3)	69 (19.3)				
>−1 to ≤1	309 (60.1)	184 (51.5)				
>1	41 (8.0)	24 (6.7)				
**DAS-II nonverbal IQ**						
≤−2	60 (11.7)	70 (19.5)	−0.31 (−0.49 to −0.12)	−0.22 (−0.39 to −0.05)	NA	NA
>−2 to ≤−1	117 (22.8)	96 (26.7)				
>−1 to ≤1	312 (60.7)	173 (48.2)				
>1	25 (4.9)	20 (5.6)				
**DAS-II full-scale IQ**						
≤−2	64 (12.5)	71 (19.9)	−0.38 (−0.56 to −0.19)	−0.29 (−0.45 to −0.12)	NA	NA
>−2 to ≤−1	85 (16.6)	79 (22.1)				
>−1 to ≤1	346 (67.4)	192 (53.8)				
>1	18 (3.5)	15 (4.2)				
**DAS-II Working Memory**						
≤−2	75 (14.7)	81 (22.6)	−0.36 (−0.56 to −0.16)	−0.26 (−0.45 to −0.08)	NA	NA
>−2 to ≤−1	86 (16.8)	67 (18.7)				
>−1 to ≤1	332 (64.8)	196 (54.8)				
>1	19 (3.7)	14 (3.9)				
**NEPSY-II Inhibition Inhibition**						
≤−2	153 (30.5)	134 (38.3)	−0.27 (−0.44 to −0.10)	−0.22 (−0.38 to −0.05)	NA	NA
>−2 to ≤−1	121 (24.1)	78 (22.3)				
>−1 to ≤1	206 (41.0)	131 (37.4)				
>1	22 (4.4)	7 (2.0)				
**NEPSY-II Inhibition Switching**						
≤−2	120 (24.3)	110 (31.9)	−0.17 (−0.35 to 0)	−0.10 (−0.26 to 0.07)	NA	NA
>−2 to ≤−1	149 (30.2)	94 (27.3)				
>−1 to ≤1	192 (39.0)	119 (34.5)				
>1	32 (6.5)	22 (6.4)				
**Neurocognitive function composite**						
Normal	181 (35.1)	119 (33.2)	NA	NA	1.57 (1.25 to 1.97)	1.40 (1.13 to 1.74)
Low normal	232 (45.0)	128 (35.8)				
Moderate	74 (14.3)	71 (19.8)				
Severe	29 (5.6)	40 (11.2)				
Autism spectrum disorder	24 (4.7)	37 (10.6)	NA	NA	2.18 (1.37 to 3.49)	1.84 (1.15 to 2.95)
Epilepsy	25 (4.8)	41 (11.2)	NA	NA	2.29 (1.42 to 3.69)	2.07 (1.31 to 3.27)
ADHD	91 (17.6)	61 (17.1)	NA	NA	0.95 (0.71 to 1.28)	0.91 (0.68 to 1.21)
Anxiety	78 (15.1)	54 (15.1)	NA	NA	1.03 (0.75 to 1.43)	0.98 (0.71 to 1.35)
Depression	35 (6.8)	24 (6.7)	NA	NA	1.10 (0.67 to 1.82)	1.10 (0.66 to 1.83)

Abbreviations: ADHD, attention-deficit/hyperactivity disorder; aRR, adjusted relative risk; DAS-II, School-Age Differential Ability Scales–II; NA, not applicable; NEPSY-II, Developmental Neuropsychological Assessment, second edition; RR, relative risk.

^a^ Adjusted for insurance, maternal education, sex, white matter damage, and chronic lung disease (multiple imputation was used for missing covariates).

Acid suppressant use was significantly associated with ASD (aRR, 1.84; 95% CI, 1.15-2.95) (Table 2). Acid suppressant use was also associated with increased scores for impaired social ability as indexed by the SRS-2 (adjusted β, 1.96; 95% CI, 0.36-3.56) SCI and RRB (adjusted β, 1.77; 95% CI, 0.18-3.35) assessment scores. Acid suppressant use was associated with epilepsy (aRR, 2.07; 95% CI, 1.31-3.27) but not ADHD, anxiety, or depression (Table 2).

### Secondary Analyses

Poisson models of acid suppressant use and ASD with or without intellectual impairment, compared with no ASD or intellectual development, identified an association with ASD with intellectual impairment (aRR, 2.14; 95% CI, 1.11-4.12), and no association was found with ASD without intellectual impairment (aRR, 1.77; 95% CI, 0.86-3.63). No association was observed with intellectual impairment alone (adjusted odds ratio [aOR], 1.25; 95% CI, 0.83-1.90) (Table 3).

**Table 3.** Acid Suppressant Use and ASD With and Without Intellectual Disability at Age 10

	Acid suppressant ≤24 mo of age, No. (%)		OR (95% CI)	aOR (95% CI)^a^
	None	Any		
No ASD and no intellectual impairment	438 (62)	269 (38)	[reference]	[reference]
No ASD and intellectual impairment	39 (53)	35 (47)	1.43 (0.93-2.20)	1.25 (0.83-1.90)
ASD and no intellectual impairment	11 (42)	15 (58)	1.99 (0.96-4.14)	1.77 (0.86-3.63)
ASD and intellectual impairment	12 (36)	21 (64)	2.67 (1.35-5.27)	2.14 (1.11-4.12)

Abbreviations: aOR, adjusted odds ratio; ASD, autism spectrum disorder.

^a^ Adjusted for sex, insurance, maternal education, white matter damage, and chronic lung disease (multiple imputation was used for missing covariates).

### Sensitivity Analyses

Results from the multinomial regression models were consistent with the results observed in the primary analyses. Of note, however, for the composite (LPA) measure of neurocognitive function, the odds of severe impairment (aOR, 2.2; 95% CI, 1.2-3.8) and low-normal impairment (aOR, 1.7; 95% CI, 1.1-2.6) were higher compared with normal neurocognitive function (eTable 2 in the Supplement). Assessment of model fit when parameterizing H2RA and PPIs independently suggested no improvement for model fit compared with the combination of H2RAs and PPIs into a single measure of acid suppressant exposure (eTable 3 in the Supplement).

The IPW analyses, jointly accounting for probability of treatment and loss to follow-up also showed associations, as observed in the primary analyses. For example, we observed a 33% higher risk of moderate to severe neurocognitive impairment relative to normal or low-normal impairment (aRR, 1.33; 95% CI, 1.04-1.68), as well as a 75% higher risk of ASD (aRR, 1.75; 95% CI, 1.07-2.87) (eTable 4 in the Supplement).

In the propensity score–matched analyses, 328 of the 521 exposed infants could be matched with an unexposed infant. Neurocognitive outcome analyses were conducted from this matched data set of 656 subjects and results were generally consistent with results obtained in the primary and IPW model approaches (eTable 5 in the Supplement).

Assessment of the association between acid suppressant use according to timing of use suggested marginal differences according to timing, although the direction of the association remained consistent across all exposure periods. Acid suppressant use in the NICU was associated with a higher risk of ASD (aRR, 1.85; 95% CI, 1.07 to 3.20), whereas neurocognitive impairment was associated with use after discharge or in the NICU and postdischarge periods combined (eTable 6 in the Supplement).

Restriction of the study participant sample to those without indication of cerebral palsy or use of a feeding tube yielded similar findings to those in the primary case analysis for both neurocognitive and neurodevelopmental outcomes (eTable 7 in the Supplement).

## Discussion

In this cohort study, among children who were born extremely preterm, we observed associations between acid suppressant use in the first 24 months of life and multiple adverse neurocognitive and neurodevelopmental outcomes at age 10 years, including lower scores on measures of IQ (equating to a decrease of 4.35 [95% CI, 6.75-1.80] points on full scale IQ), EF, and working memory and higher likelihoods of ASD and epilepsy. Although the specific mechanisms underlying these associations are uncertain, acid suppressant use has been associated with changes in the intestinal microbiome and dysbiosis,^44^ which in turn has been associated with neurocognitive outcomes. In a study of infants,^45^ use of a PPI decreased the relative abundance of *Lactobacillus* and *Stenotrophomonas* species and increased the relative abundance of *Haemophilus* strains. Intestinal dysbiosis in the preterm infant, during this period of physiological stress, may be particularly detrimental to neurodevelopment. In a murine model, administering acid suppressants in the setting of acute illness resulted in differential expression of genes involved in behavioral and cognitive pathways^23^; this differential expression was correlated with the relative abundance of microbial families. Fecal microbial transfer treatments have been shown to decrease behavioral symptoms of ASD,^44^ although evidence relating gut microbiome composition to ASD remains mixed, likely due to the need for more sophisticated study designs and analytic methods.^46^ Of note, acid suppressants have been associated with alterations to intestinal barrier function and integrity,^47^ particularly during physiological stress,^25,48^ and children with ASD have been identified as having altered intestinal barrier function.^49,50,51^ Still, the temporality of these associations has not been established.

Data from adult populations suggest that PPI use is significantly associated with a wide range of neurological outcomes, including migraines, peripheral neuropathies, and visual and auditory neurosensory abnormalities.^26^ Multiple mechanisms suggested include PPI associations with immune cell activation, migration, and function; epithelial cell signaling by inhibiting interleukin 8 transcription; and reduction of endothelial nitric oxide synthetase activity.^52^ These mechanisms suggest biological plausibility for a link between acid suppressants and preterm infant neurodevelopment. Of note, PPI-associated adverse outcomes have been found more frequently among individuals with a specific variant in the *CYP2C19* gene, which influences PPI metabolism.^19^ Future investigations may explore possible heterogeneity of effects according to this variant.

### Limitations

This study has limitations. The observed results were sensitive to analyses evaluating impairment severity and possible bias both confounding by indication and loss to follow-up; however, our inverse probability weighted models assumed loss to follow-up was missing at random and that we have adequately modeled factors associated with loss to follow-up and probability of treatment. Our propensity score–matched analysis also assumes we have adequately modeled propensity for treatment and that we are not missing factors associated with treatment. We included in these models indication of feeding difficulties, but other factors could contribute to use of these medications in early life. Still, bias would likely only result if there were unaccounted factors associated with treatment and neurodevelopmental outcomes. Misclassification of acid suppressant use is possible, given the potential for missing data with abstraction of medical records (for use during NICU admission) and reliance on maternal report of medication use in the postdischarge period. Nevertheless, this would likely result in underreporting of use and an attenuation of association estimates observed. It is also possible that the associations observed could be attributable to unmeasured confounding. Although we adjusted for both white matter damage indicated by ultrasonographic scans and maternal report of early-life feeding difficulties, the specific indication for acid suppressant use was not recorded, and it is plausible that other infant behaviors indicative of infant neurological dysfunction and dysregulation could have led to treatment with acid suppressants. Still, the results from our sensitivity analysis, which included restriction of our sample to infants without cerebral palsy and without a feeding tube, as well as other sensitivity analyses designed to address potential assumptions in our primary analyses, were all generally consistent with our primary analysis results. Although we examined exposure according to timing, we were not able to measure precise exposure periods or cumulative dosages; thus, differences observed between timing could also be attributable to differences in duration of use or simply the smaller sample sizes for the subanalyses.

The present study has implications for the possible adverse effects of acid suppressants on neurodevelopment in children born extremely preterm, who generally experience intense physiological stress and may thus be particularly sensitive to these medications. Although there is increased awareness of the lack of benefit for use of these agents in the NICU, postdischarge use remains frequent.^8,53,54,55^

## Conclusions

The results of this cohort study examining early-life acid suppressants use in relation to neurocognitive and neurodevelopmental outcomes in children who were born extremely preterm add to the growing evidence of potential risks of long-term adverse outcomes from use of acid suppressants in early life, particularly among extremely preterm infants. Given the frequent use of these agents in this population, further investigation into the observed associations is warranted.

## Notes

Supplement. **eTable 1.** Participant Characteristics at Baseline and Age 10 Years: The ELGAN Cohort Study (2002-2020) **eTable 2.** Acid Suppression and Neurocognitive and Neurodevelopmental Outcomes by Outcome Severity at Age 10: The ELGAN Cohort Study (2002-2020) **eTable 3.** Likelihood Ratio Test for Model Selection Comparing PPI and H2RA Combined vs PPI and H2RA as Separate Covariates: The ELGAN Cohort Study (2002-2020) **eTable 4.** Acid Suppressant Use and Neurocognitive, Neurodevelopmental, and Psychiatric Outcomes at Age 10 With Inverse Probability Weighting: The ELGAN Cohort Study (2002-2020) **eTable 5.** Propensity Score Matching Analysis of Acid Suppressant Exposure at Age 10: The ELGAN Cohort Study (2002-2020) **eTable 6.** Acid Suppressant Use According to Timing of Exposure and Neurodevelopmental Outcomes: The ELGAN Cohort Study (2002-2020) **eTable 7.** Acid Suppressant Use Within Subset of Participants Without Cerebral Palsy and Without Tube Feedings: The ELGAN Cohort Study (2002-2020) **eFigure.** Directed Acyclic Graph
