# Supplementary material for: Analysis of Neurodevelopment in Children Born Extremely Preterm Treated With Acid Suppressants Before Age 2 Years
Source: JAMA Netw Open. 2022 Nov 15;5(11):e2241943. doi: 10.1001/jamanetworkopen.2022.41943 (PMC9667324; doi:10.1001/jamanetworkopen.2022.41943)
Supplement: Supplement. — eTable 1. Participant Characteristics at Baseline and Age 10 Years: The ELGAN Cohort Study (2002-2020) eTable 2. Acid Suppression and Neurocognitive and Neurodevelopmental Outcomes by Outcome Severity at Age 10: The ELGAN Cohort Study (2002-2020) eTable 3. Likelihood Ratio Test for Model Selection Comparing PPI and H2RA Combined vs PPI and H2RA as Separate Covariates: The ELGAN Cohort Study (2002-2020) eTable 4. Acid Suppressant Use and Neurocognitive, Neurodevelopmental, and Psychiatric Outcomes at Age 10 With Inverse Probability Weighting: The ELGAN Cohort Study (2002-2020) eTable 5. Propensity Score Matching Analysis of Acid Suppressant Exposure at Age 10: The ELGAN Cohort Study (2002-2020) eTable 6. Acid Suppressant Use According to Timing of Exposure and Neurodevelopmental Outcomes: The ELGAN Cohort Study (2002-2020) eTable 7. Acid Suppressant Use Within Subset of Participants Without Cerebral Palsy and Without Tube Feedings: The ELGAN Cohort Study (2002-2020) eFigure. Directed Acyclic Graph [file jamanetwopen-e2241943-s001.pdf]

Supplemental Online Content

Jensen ET, Yi J, Jackson W, et al. Analysis of neurodevelopment in children born extremely preterm treated with acid suppressants before age 2 years. *JAMA Netw Open*. 2022;5(11):e2241943. doi:10.1001/jamanetworkopen.2022.41943

eTable 1. Participant Characteristics at Baseline and Age 10 Years: The ELGAN Cohort Study (2002-2020)

eTable 2. Acid Suppression and Neurocognitive and Neurodevelopmental Outcomes by Outcome Severity at Age 10: The ELGAN Cohort Study (2002-2020)

eTable 3. Likelihood Ratio Test for Model Selection Comparing PPI and H2RA Combined vs PPI and H2RA as Separate Covariates: The ELGAN Cohort Study (2002-2020)

eTable 4. Acid Suppressant Use and Neurocognitive, Neurodevelopmental, and Psychiatric Outcomes at Age 10 With Inverse Probability Weighting: The ELGAN Cohort Study (2002-2020)

eTable 5. Propensity Score Matching Analysis of Acid Suppressant Exposure at Age 10: The ELGAN Cohort Study (2002-2020)

eTable 6. Acid Suppressant Use According to Timing of Exposure and Neurodevelopmental Outcomes: The ELGAN Cohort Study (2002-2020)

eTable 7. Acid Suppressant Use Within Subset of Participants Without Cerebral Palsy and Without Tube Feedings: The ELGAN Cohort Study (2002-2020)

eFigure. Directed Acyclic Graph

This supplemental material has been provided by the authors to give readers additional information about their work.

© 2022 Jensen ET et al. *JAMA Network Open.*

eTable 1. Participant Characteristics at Baseline and Age 10 Years: The ELGAN Cohort Study (2002-2020)

Characteristic	Enrolled at birth (n=1506)	Survived to 24 months (n=1198)	Age 10 years (n=889)
Gestational age at delivery - mean (SD)	25.9 (1.3)	26.1 (1.3)	26.1 (1.3)
Sex – n (%)			
Male	799 (53.0%)	621 (51.8%)	455 (51.2%)
Female	707 (47.0%)	577 (48.2%)	434 (48.8%)
Weight for gestational age at delivery z-score - mean (SD)	-0.27 (1.12)	-0.16 (1.06)	-0.19 (1.09)
Chronic lung disease – n (%)			
Neither O2 nor Ventilator at 36 weeks	597 (48.1%)	590 (49.7%)	421 (47.7%)
O2 at 36 weeks	520 (41.9%)	498 (41.9%)	380 (43.1%)
Ventilator at 36 weeks	124 (10.0%)	100 (8.4%)	81 (9.2%)
White matter damage – n (%)	347 (23.9%)	242 (20.2%)	188 (21.2%)
Maternal race – n (%)			
Black	427 (28.9%)	322 (27.3%)	227 (25.6%)
White	866 (58.5%)	706 (59.9%)	562 (63.4%)
Other	187 (12.7%)	151 (12.8%)	98 (11.1%)
Maternal ethnicity – n (%)			
Hispanic	179 (12.0%)	147 (12.3%)	86 (9.7%)
Non-Hispanic	1313 (88.0%)	1045 (87.7%)	800 (90.3%)
Maternal education (years) at Birth – n (%)			
≤12	633 (45.2%)	506 (43.9%)	355 (41.1%)
>12 - <16	340 (24.3%)	270 (23.4%)	202 (23.4%)
≥16	428 (30.6%)	376 (32.6%)	306 (35.5%)
Public insurance – n (%)			
No	841 (58.6%)	709 (60.4%)	568 (64.9%)
Yes	594 (41.4%)	464 (39.6%)	307 (35.1%)
Feeding difficulties at 12 months – n (%)			
Missing*	481 (31.9%)	179 (14.9%)	93 (10.5%)
No	749 (49.7%)	746 (62.3%)	574 (64.6%)
Yes	276 (18.3%)	273 (22.8%)	222 (25.0%)
Cerebral Palsy – n (%)**			
Quad	64 (6.1%)	63 (6.0%)	48 (5.7%)
Di	37 (3.5%)	37 (3.5%)	29 (3.4%)
Hemi	19 (1.8%)	19 (1.8%)	16 (1.9%)
None	936 (88.6%)	936 (88.7%)	756 (89.1%)

*Participants that died prior to the 12 month follow-up visit have this data missing

**Collected at 24 months of age

eTable 2. Acid Suppression and Neurocognitive and Neurodevelopmental Outcomes by Outcome Severity at Age 10: The ELGAN Cohort Study (2002-2020)

	Crude OR (95% CI)			Adjusted OR (95% CI)		
Outcome	Impaired (≤ −2)	Low Normal (−2 < − ≤ −1)	Referent > -1	Impaired (≤ −2)	Low Normal (−2 < − ≤ −1)	Referent > -1
DAS verbal IQ	2.0 (1.3, 2.8)	1.2 (0.9, 1.8)	Referent	1.8 (1.2, 2.7)	1.3 (0.9, 1.9)	Referent
DAS non-verbal IQ	2.0 (1.4, 3.0)	1.4 (1.0, 2.0)	Referent	1.9 (1.3, 2.9)	1.5 (1.0, 2.1)	Referent
DAS Full Scale IQ	2.0 (1.4, 2.9)	1.6 (1.1, 2.3)	Referent	1.8 (1.2, 2.8)	1.8 (1.2, 2.6)	

© 2022 Jensen ET et al. *JAMA Network Open.*

Neurocognitive function composite	2.5 (1.5, 4.2)	1.7 (1.2, 2.6)	Referent	2.2 (1.2, 3.8)	1.7 (1.1, 2.6)	Referent
DAS working memory	1.8 (1.2, 2.6)	1.3 (0.9, 1.9)	Referent	1.7 (1.2, 2.6)	1.3 (0.9, 1.9)	Referent
NEPSY inihibition inhibition	1.3 (1.0, 1.8)	1.0 (0.7, 1.4)	Referent	1.3 (0.9, 1.8)	1.0 (0.7, 1.5)	Referent
NEPSY inhibition switching	1.4 (1.0, 2.0)	1.0 (0.7, 1.4)	Referent	1.4 (1.0, 2.0)	1.0 (0.7, 1.4)	Referent
SCI (DSM-5 Compatible Scores)	1.1 (0.5, 2.1)	0.7 (0.4, 1.2)	Referent	0.9 (0.5, 1.9)	0.7 (0.4, 1.2)	Referent
RRB (DSM-5 Compatible Scores)	1.6 (0.8, 3.1)	1.2 (0.6, 2.3)	Referent	1.6 (0.8, 3.1)	1.1 (0.6, 2.1)	Referent

*Adjusted for sex, insurance, maternal education, white matter damage, and chronic lung disease (missing covariates were multiple imputed)

eTable 3. Likelihood Ratio Test for Model Selection Comparing PPI and H2RA Combined vs PPI and H2RA as Separate Covariates: The ELGAN Cohort Study (2002-2020)

	Full -2 Log Likelihood (PPI only + H2RA Only)	Reduced Model -2 Log Likelihood (PPI and H2RA combined)	Chi-Square	df	p-value
Neurocognitive function composite	976.9904	976.1424	0.848	1	0.3571
Autism	394.8026	394.7584	0.0442	1	0.8335
ADHD	767.3418	767.4678	0.126	1	0.7226
Generalized Anxiety	719.1986	719.8618	0.6632	1	0.4154
Depression	398.894	398.4812	0.4128	1	0.5206

eTable 4. Acid Suppressant Use and Neurocognitive, Neurodevelopmental, and Psychiatric Outcomes at Age 10 With Inverse Probability Weighting: The ELGAN Cohort Study (2002-2020)

	aRR (95% CI)
Neurocognitive function composite	
Normal or low normal	Referent
Moderate or severe	1.33 (1.04, 1.68)
Autism	
No	Referent

© 2022 Jensen ET et al. *JAMA Network Open.*

Yes	1.75 (1.07, 2.87)
Epilepsy	
No	Referent
Yes	1.97 (1.19, 3.24)
ADHD	
No	Referent
Yes	0.88 (0.65, 1.19)
Anxiety	
No	Referent
Yes	0.99 (0.72, 1.38)
Depression	
No	Referent
Yes	1.10 (0.65, 1.84)

*Adjusted for sex, insurance, maternal education, white matter damage, and chronic lung disease

eTable 5. Propensity Score Matching Analysis of Acid Suppressant Exposure at Age 10: The ELGAN Cohort Study (2002-2020)

Outcome	Level	RR	aRR	Parametric P-value *
DAS verbal IQ	≤ -2	1.58 (1.06, 2.36)	1.68 (1.08, 2.61)	0.0215
	> -2, ≤ -1	1.31 (0.87, 1.97)	1.34 (0.88, 2.05)	0.1664
	>-1, ≤ 1			
	>1	1.18 (0.64, 2.16)	1.17 (0.62, 2.18)	0.6291
DAS non-verbal IQ	≤ -2	1.69 (1.10, 2.59)	1.74 (1.09, 2.75)	0.0191
	> -2, ≤ -1	1.36 (0.94, 1.96)	1.38 (0.94, 2.01)	0.0962
	>-1, ≤ 1			
	>1	1.54 (0.77, 3.11)	1.53 (0.75, 3.14)	0.2419
DAS full scale IQ	≤ -2	1.61 (1.07, 2.44)	1.69 (1.08, 2.66)	0.0223
	> -2, ≤ -1	1.66 (1.10, 2.50)	1.70 (1.11, 2.59)	0.0138
	>-1, ≤ 1			
	>1	1.47 (0.67, 3.22)	1.40 (0.62, 3.14)	0.4134
Neurocognitive function composite	Normal			
	Low normal	0.69 (0.48, 0.98)	0.69 (0.48, 1.00)	0.0529

© 2022 Jensen ET et al. *JAMA Network Open.*

	Moderate	1.16 (0.74, 1.81)	1.18 (0.73, 1.92)	0.4979
	Severe	1.37 (0.78, 2.42)	1.39 (0.74, 2.61)	0.3124
DAS working memory	≤ -2	1.40 (0.94, 2.09)	1.43 (0.93, 2.22)	0.1058
	> -2, ≤ -1	1.23 (0.82, 1.84)	0.88 (0.40, 1.93)	0.7558
	>-1, ≤ 1			
	>1	0.92 (0.43, 1.99)	1.13 (0.50, 2.59)	0.7641
NEPSY inihibition inhibition	≤ -2	1.06 (0.74, 1.52)	1.05 (0.73, 1.53)	0.7821
	> -2, ≤ -1	0.92 (0.61, 1.39)	0.92 (0.61, 1.39)	0.6902
	>-1, ≤ 1			
	>1	0.41 (0.16, 1.03)	0.40 (0.16, 1.03)	0.0571
NEPSY inhibition switching	≤ -2	1.14 (0.78, 1.69)	1.11 (0.73, 1.68)	0.6257
	> -2, ≤ -1	0.89 (0.61, 1.32)	0.87 (0.58, 1.30)	0.4986
	>-1, ≤ 1			
	>1	1.06 (0.55, 2.05)	1.05 (0.54, 2.04)	0.8799
SCI (DSM-5 Compatible Scores)				
	Normal			
	Mild	1.73 (1.03, 2.91)	1.69 (1.00, 2.85)	0.0497
	Moderate	1.07 (0.68, 1.70)	1.05 (0.65, 1.68)	0.8394
	Severe	1.70 (0.89, 3.25)	1.75 (0.90, 3.40)	0.0961
RRB (DSM-5 Compatible Scores)				
	Normal			
	Mild	1.01 (0.59, 1.74)	1.03 (0.60, 1.78)	0.9150
	Moderate	1.18 (0.70, 1.98)	1.13 (0.66, 1.93)	0.6572
	Severe	1.64 (0.96, 2.82)	1.65 (0.96, 2.85)	0.0710
Working Memory z-score ≤ -1		1.20 (0.99, 1.45)	1.17 (0.97, 1.40)	0.0952
Inhibition Inhibition z-score ≤ -1		1.10 (0.90, 1.34)	1.07 (0.88, 1.30)	0.4837
Inhibition Switching z-score ≤ -1		1.14 (0.90, 1.44)	1.10 (0.88, 1.38)	0.3944
Autism spectrum disorder	%	1.76 (1.06, 2.93)	1.71 (1.04, 2.83)	0.0361
Epilepsy	%	2.27 (1.31, 3.96)	2.17 (1.28, 3.66)	0.0037

© 2022 Jensen ET et al. *JAMA Network Open.*

ADHD	%	0.86 (0.63, 1.18)	0.87 (0.64, 1.19)	0.3859
Anxiety (parent report)	%	1.00 (0.71, 1.43)	0.99 (0.69, 1.41)	0.9409
Depression (parent report*)*	%	1.13 (0.64, 2.01)	1.17 (0.65, 2.08)	0.6048

eTable 6. Acid Suppressant Use According to Timing of Exposure and Neurodevelopmental Outcomes: The ELGAN Cohort Study (2002-2020)

	None	NICU Only<br>Beta (95% CI)	Post Discharge Only<br>Beta (95% CI)	NICU + Post Discharge Only<br>Beta (95% CI)
DAS-II verbal IQ z-score	Referent	-0.19 (-0.41, 0.04)	-0.53 (-0.82,-0.23)	-0.46 (-0.85,-0.07)
DAS-II non-verbal IQ z-score	Referent	-0.06 (-0.26, 0.15)	-0.48 (-0.76,-0.20)	-0.29 (-0.62, 0.04)
DAS-II Full Scale IQ z-score	Referent	-0.13 (-0.34, 0.07)	-0.50 (-0.77,-0.23)	-0.39 (-0.72,-0.06)
DAS-II Working Memory z-score	Referent	-0.12 (-0.36, 0.11)	-0.40 (-0.67,-0.12)	-0.47 (-0.87,-0.06)
NEPSY-II Inhibition Inhibition z-score	Referent	-0.21 (-0.41,-0.01)	-0.34 (-0.59,-0.10)	-0.12 (-0.46, 0.22)
NEPSY-II Inhibition Switching z-score	Referent	-0.09 (-0.29, 0.12)	-0.24 (-0.49, 0.00)	-0.09 (-0.44, 0.27)
		aRR (95% CI)	aRR (95% CI)	aRR (95% CI)
Neurocognitive function composite (LPA)	Referent	1.24 (0.93,1.66)	1.60 (1.24,2.08)	1.45 (0.98,2.16)
Autism spectrum disorder	Referent	1.85 (1.07,3.20)	1.66 (0.90,3.09)	1.56 (0.70,3.49)
Epilepsy	Referent	1.64 (0.91,2.96)	2.46 (1.39,4.35)	1.99 (0.89,4.44)
ADHD	Referent	0.85 (0.59,1.22)	0.94 (0.61,1.44)	1.02 (0.59,1.76)
Anxiety	Referent	0.92 (0.61,1.38)	1.17 (0.75,1.83)	1.23 (0.67,2.28)
Depression	Referent	0.65 (0.31,1.38)	2.41 (1.32,4.42)	0.66 (0.17,2.57)

Adjusted Beta Coefficients and 95% CI for Verbal IQ, non-verbal IQ, full scale IQ, working memory, inhibition inhibition, inhibition switching

Adjusted RR and 95% CI for Neurocognitive function composite, Autism spectrum disorder, epilepsy, ADHD, Anxiety, Depression

*Adjusted for sex, insurance, maternal education, white matter damage, and chronic lung disease (missing covariates were multiple imputed)

© 2022 Jensen ET et al. *JAMA Network Open.*

eTable 7. Acid suppressant use within subset of participants without cerebral palsy and without tube feedings: The ELGAN Cohort Study (2002-2020)

	No Cerebral Palsy<br><br>Beta (95% CI)	No Tube Feeding<br><br>Beta (95% CI)
DAS-II verbal IQ	-0.32 (-0.50,-0.14)	-0.27 (-0.47,-0.08)
DAS-II non-verbal IQ	-0.19 (-0.36,-0.03)	-0.21 (-0.40,-0.03)
DAS-II Full Scale IQ	-0.26 (-0.41,-0.10)	-0.25 (-0.43,-0.07)
DAS-II Working Memory	-0.21 (-0.39,-0.03)	-0.26 (-0.45,-0.06)
NEPSY-II Inhibition Inhibition	-0.25 (-0.42,-0.08)	-0.18 (-0.36,-0.01)
NEPSY-II Inhibition Switching	-0.08 (-0.26, 0.09)	-0.07 (-0.25, 0.11)
	aRR (95% CI)	aRR (95% CI)
Neurocognitive function composite (LPA)	1.45 (1.11,1.90)	1.31 (1.02,1.69)
Autism spectrum disorder	1.96 (1.19,3.23)	1.83 (1.10,3.03)
Epilepsy	2.78 (1.55,4.98)	2.37 (1.42,3.94)
ADHD	0.94 (0.69,1.27)	0.97 (0.71,1.33)
Anxiety	0.96 (0.69,1.34)	0.95 (0.66,1.34)
Depression	1.05 (0.61,1.80)	0.92 (0.53,1.58)

*Adjusted for sex, insurance, maternal education, white matter damage, and chronic lung disease (missing covariates were multiple imputed)

© 2022 Jensen ET et al. *JAMA Network Open.*

eFigure. Directed Acyclic Graph

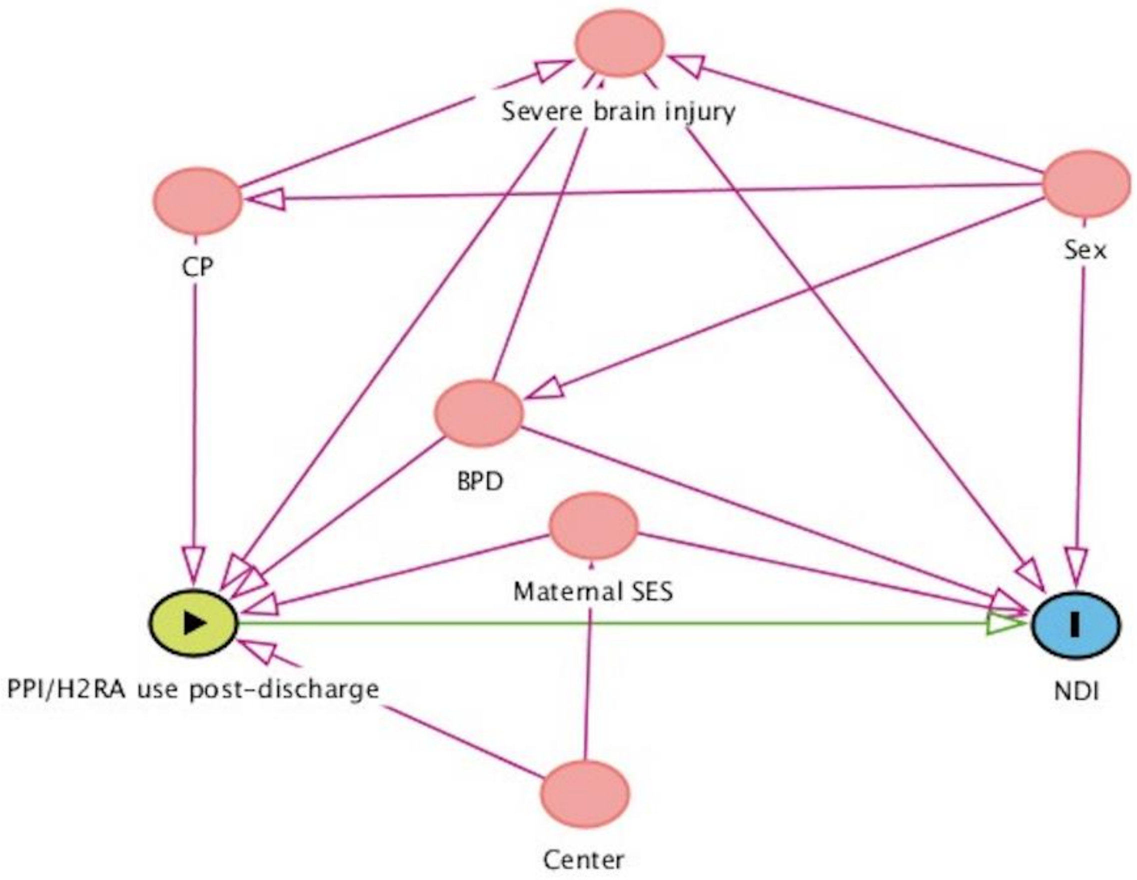

© 2022 Jensen ET et al. *JAMA Network Open.*
